# Case of Irreducible Scrotal Hernia Successfully Treated

**Published:** 1861-04

**Authors:** Ross C. Russ

**Affiliations:** Royalton, Ind.


### CASE OF IRREDUCIBLE SCROTAL HERNIA SUCCESSFULLY TREATED.

BY ROSS C. RUSS, M. D., Royalton, Ind.

In August, 1859, I was summoned to visit Silas Crane, a lad eight years of age, suffering with scrotal hernia. The usual taxis was employed without success. In the absence of any evidence of strangulation of the intestine, it was deemed unnecessary to operate. Directed the patient to be placed in the dorsal position, with the shoulders and pelvis raised so as relax the abdominal muscles. This position was retained three weeks. The bowels were kept open by mild cathartics, opiates to procure rest at night and relieve local irritation, lotions of Acet. Plumbi externally to the scrotum. At the expiration of three weeks I was again called; found the tumor much smaller, and by very little manipulation the knuckle of bowel was easily reduced.

One of Marsh's radical cure trusses was then applied, and up to the present date (Feb. 15th, 1861) there has been no return of the difficulty.

